# Dietary Salt Restriction Practices Contribute to Obesity Prevention in Middle-Aged and Older Japanese Adults

**DOI:** 10.3390/nu17030536

**Published:** 2025-01-31

**Authors:** Etsuko Kibayashi, Makiko Nakade

**Affiliations:** 1 Department of Food and Nutrition, Sonoda Women’s University, Amagasaki 661-8520, Hyogo, Japan; 2 Department of Food Science and Nutrition, University of Hyogo, Himeji 670-0092, Hyogo, Japan; 3 Research Institute for Food and Nutritional Sciences, Himeji 670-0092, Hyogo, Japan

**Keywords:** middle-aged and older Japanese adults, salt restriction, Na/K ratio, body mass index, obesity prevention

## Abstract

**Background/Objectives**: In a demographic increasingly at risk of obesity and hypertension, whether dietary behaviours associated with hypertension prevention, such as restricting salt intake and consuming potassium-rich fruits and vegetables, contribute to obesity prevention is unclear. Therefore, we aimed to investigate the structural associations of dietary salt restriction practices with body mass index (BMI) and the mediating role of the dietary sodium/potassium (Na/K) ratio in middle-aged and older Japanese adults. **Methods**: This cross-sectional survey included 418 participants aged 40–69 years, residing in Hyogo, Japan. Simultaneous multi-population analysis according to sex was performed using a hypothetical model to explore associations of dietary salt restriction practices with BMI, mediated by the dietary Na/K ratio, as well as the role of BMI-related eating behaviours. **Results:** Dietary salt restriction was associated with a low dietary Na/K ratio (standardised estimate: –0.21, *p* = 0.001 for men; –0.19, *p* = 0.002 for women) in both sexes. Dietary salt restriction was associated with lower values of BMI in men (0.21, *p* = 0.004), mediated by the Na/K ratio; a direct but relatively weak association with lower values of BMI was observed in women (−0.16, *p* = 0.018). In men, eating out frequently was associated with higher values of BMI (0.20, *p* = 0.005). **Conclusions**: Dietary salt restriction practices in middle-aged and older adults may contribute to obesity prevention, and dietary Na/K ratio may play a mediating role in men but not in women; additionally, eating out was associated with higher values of BMI in men.

## 1. Introduction

The World Health Organization (WHO) reports that reducing sodium intake is one of the most cost-effective measures to reduce the burden of non-communicable diseases [1]. The national health promotion policy of Japan, ‘Health Japan 21 (third stage)’, also recommends reducing salt intake [2]. A reduction in dietary salt intake estimated from 24 h urinary sodium excretion has been shown to reduce blood pressure [3] and the risk of cardiovascular disease (CVD) [4]. An increase in dietary potassium intake estimated from 24 h urinary potassium excretion has been shown to reduce blood pressure [5] and CVD-related mortality [6]. Therefore, preventing hypertension is crucial for preventing CVD development. The Guidelines for the Management of Hypertension 2019 of the Japanese Society of Hypertension [7] recommend a reduced intake of salt and an increased intake of fruits and vegetables. An intervention study using the Dietary Approaches to Stop Hypertension diet [8] revealed that increased potassium intake had an even greater reduction effect on blood pressure in addition to salt reduction. In a study based in the United States, the dietary sodium/potassium (Na/K) ratio was found to be associated with CVD-related and all-cause mortality when calculated using data from 24 h dietary recalls [9] and with all-cause mortality when using data from food frequency questionnaires [10]. Furthermore, in a study involving Japanese individuals, the Na/K ratio was shown to be a risk factor for stroke and CVD when calculated from 3-day weighed food record data [11].

The association between obesity and CVD [12] is commonly reported among various diseases involving dyslipidaemia, hyperglycaemia, and hypertension, given that obesity is a risk factor for atherosclerosis. Obesity leads to the development of CVD and CVD-related mortality [13]. Cohort studies have demonstrated that a high BMI [14,15] and increased body weight [16,17] are risk factors for hypertension. Additionally, salt intake estimated from 24 h urinary sodium excretion has been positively correlated with BMI and overweight/obesity rates in Japan, China, the United Kingdom, and the United States [18]. Accordingly, in overweight and obese individuals, dietary salt restriction is often required to prevent hypertension. In contrast, consuming vegetables and fruits with high potassium levels increases satiety, suppresses hunger, and reduces energy intake. Although vegetable and fruit intake is negatively associated with the risk of CVD-related mortality [19], there is insufficient evidence on whether it causes weight loss [20]. Nonetheless, in a demographic increasingly at risk of obesity and hypertension, dietary behaviours associated with hypertension prevention, such as restricting salt intake and consuming potassium-rich fruits and vegetables, are expected to contribute to obesity prevention. Therefore, the impact of dietary salt restriction practices on weight loss and obesity prevention should be further explored in conjunction with the association of other dietary behaviours, including salt, sodium, and potassium intakes, with hypertension prevention.

Several studies have investigated eating and lifestyle behaviours related to BMI and overweight/obesity. The percentage of energy intake from snacking has been significantly associated with a high BMI in working adults in Minnesota, the United States [21]. Japanese women who consumed late dinners or bedtime snacks have shown an increased risk of overweight/obesity [22]. Moreover, harmful alcohol consumption, including binge drinking, has been associated with an increased BMI in Irish adults [23] and German men [24]. Additionally, the frequency of fast-food consumption and sit-down restaurant attendance has been positively correlated with BMI in the Survey of Health of Wisconsin, the United States [25], and a high frequency of eating out among Korean adults who are overweight has been observed [26]. Exercise interventions can reduce weight, BMI, and accumulated visceral fat in individuals who are overweight or obese [27]. Since various eating and lifestyle behaviours are associated with BMI and overweight/obesity, it is necessary to comprehensively examine these factors and dietary behaviours related to hypertension prevention to determine their impact on weight loss and obesity prevention.

This study aimed to investigate the structural association of dietary salt restriction practices with obesity prevention, considering the mediating role of dietary Na/K ratio, and eating behaviours related to BMI in middle-aged and older Japanese adults.

## 2. Materials and Methods

### 2.1. Study Design and Participants

This study examined secondary data from the 2021 Hyogo Nutrition and Diet Survey [28] and was approved by the Hyogo Prefectural Government. Informed consent from participants was not required owing to the use of secondary anonymised data. Since Hyogo Prefecture has been working on improving the dietary environment, especially dietary salt reduction, ahead of other prefectures, this study used data from Hyogo Prefecture; we believe that our findings based on these data can inform future dietary environment improvement measures. The survey participants (2559 household members, including 2259 individuals aged >20 years) came from 1110 households in 19 stratified, randomly selected districts in Hyogo Prefecture, based on the stratification criteria of the 2015 Population Census of Japan [29]. The data were provided by the Health Promotion Division of the Hyogo Prefectural Government in an anonymised form for secondary analysis. Ethical review was not required because the anonymised secondary data could not be linked and were not within the scope of the ethical guidelines for medical research involving human participants [30].

The survey was based on the 2019 National Health and Nutrition Survey [31]. A dietary questionnaire was distributed to each household; the questionnaire responses were collected at the same time along with those for a 66-item self-reported food frequency questionnaire (short-FFQ). The validity of the short-FFQ compared with that of the 3-consecutive-day weighed food record over four seasons was confirmed in a previous study [32]. In all, 933 individuals (41.3% response rate) responded to the dietary questionnaire (age > 20) and 929 (41.1% response rate) to the short-FFQ (age > 20).

### 2.2. Measures

We analysed the survey items obtained from the dietary questionnaire and the energy/nutrient and food group intakes calculated from the short-FFQ. Data on participant characteristics, including sex, age, living arrangement (living alone/married couple/parent(s) and children/3- or 4-generation household/others), and BMI, were collected from the dietary questionnaire. Additionally, information on the daily number of vegetable dishes (five dishes or more; four dishes; three dishes; two dishes; one or no dish) was collected, and the intake of each vegetable and fruit (g/1000 kcal) was calculated according to the density method using data from the short-FFQ. BMI, calculated as body weight (kg)/height (m)^2^ based on self-reported measurements, was classified into <18.5, 18.5–25, and ≥25 kg/m^2^. Moreover, regarding obesity, participants were classified as having obesity (≥25 kg/m^2^) or as being normal weight/underweight (<25 kg/m^2^).

The dietary salt restriction practice status was determined by the response to the question, ‘To what extent do you practise not taking in too much salt (salt reduction) to prevent or improve lifestyle-related diseases?’ Respondents were asked to select one of four response options for their dietary salt restriction practice status: ‘4 = always practising’, ‘3 = practising’, ‘2 = not practising much’, or ‘1 = not practising at all’. Additionally, ‘always practising’ + ‘practising’ and ‘not practising much’ + ‘not practising at all’ were combined into the ‘practising’ and ‘not practising’ categories, respectively, to allow comparison according to dietary salt restriction practices.

Diet-related factors included BMI as an energy intake assessment index; protein, fat, and carbohydrate/energy ratios; sodium and potassium (mg/1000 kcal), salt, vegetable, and fruit (g/1000 kcal) intakes, determined using the density method; and the dietary Na/K ratio, calculated by dividing the sodium and potassium intake by their respective atomic weights (23 and 39.1). All variables were calculated using data from the short-FFQ.

Eating and lifestyle behaviours related to BMI and their interval scales were defined as follows: snacking frequency (5 = at least twice daily; 4 = six or seven days/week; 3 = four or five days/week; 2 = two or three days/week; 1 = one day/week or less), late-night snacking frequency (5 = at least twice daily; 4 = six or seven days/week; 3 = four or five days/week; 2 = two or three days/week; 1 = one day/week or less), drinking (alcohol) frequency (7 = daily; 6 = five or six days/week; 5 = three or four days/week; 4 = one or two days/week; 3 = one to three days/month; 2 = quit; 1 = hardly drink), eating out frequency (7 = at least twice daily; 6 = once daily; 5 = four to six days/week; 4 = two or three days/week; 3 = one day/week; 2 = one to three days/month; 1 = not at all), frequency of home meal replacement consumption (i.e., the use of ready-to-eat foods; 7 = at least twice daily; 6 = once daily; 5 = four to six days/week; 4 = two or three days/week; 3 = one day/week; 2 = one to three days/month; 1 = not at all), and exercise regularity, including walking ≥30 min (5 = at least three times/week for ≥1 year; 4 = two times/week for >1 year; 3 = at least two times/week for <1 year; 2 = almost never; 1 = not at all).

### 2.3. Statistical Analysis

We analysed the data of 418 participants (190 men and 228 women) aged 40–69 years who responded to the dietary questionnaire and short-FFQ of the 2021 Hyogo Nutrition and Diet Survey. Those who had missing data on relevant items were excluded (valid response rate of 83.4% for the data of this study). Sex-based comparisons of the ratios in terms of age group, living arrangement, BMI, and daily number of vegetable dishes consumed were performed using the chi-squared test, while sex-based comparisons of vegetable and fruit intake were performed using the Mann–Whitney U test. Comparisons of BMI- and diet-related factors according to the presence of dietary salt restriction practices and sex-based comparisons of eating and lifestyle behaviour variables related to BMI were performed using Welch’s *t*-test because they were analysed on an interval scale.

**Figure 1:**
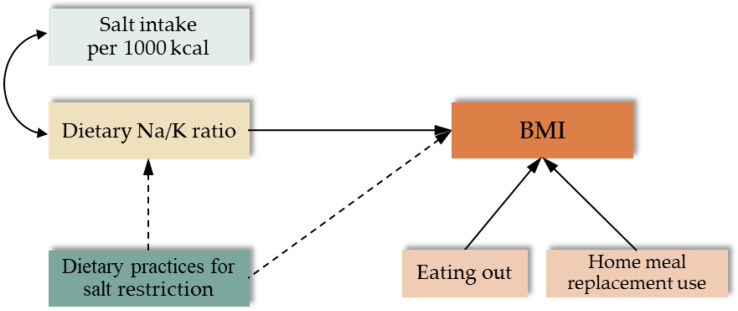
Initial hypothesised model of factors involved in dietary salt restriction practices. Two-way arc arrows indicate associations, solid arrows indicate positive pathways, and dashed arrows indicate negative pathways. BMI, body mass index; Na/K, sodium/potassium ratio.

An initial hypothetical model was developed to investigate the structural associations of dietary salt restriction practices with BMI, mediated by the dietary Na/K ratio and dietary salt intake (per 1000 kcal), including eating behaviours (eating out and home meal replacement use) related to BMI (Figure 1).

The hypothesised model was initially assessed by performing a structural analysis of covariance as part of men’s and women’s overall and structural examination. Subsequently, a simultaneous multi-population analysis according to sex was conducted to assess the model’s goodness of fit in each population and to examine construct invariance (to ascertain whether the model structure was consistent across groups) [33]. Sex-based differences in the estimates were evaluated through a pairwise comparison of parameters. We iteratively modified the model based on the path direction, standardised estimates, and fit indices, such as the goodness-of-fit index (GFI), adjusted GFI (AGFI), comparative fit index (CFI), root mean square error of approximation (RMSEA), and Akaike’s information criterion (AIC), until the optimal fit was achieved (e.g., by deleting non-significant paths). The goodness of fit of the model was determined when the GFI, AGFI, and CFI indices were >0.9, and the RMSEA was ≤0.05.

The sample size was calculated using the RMSEA for the null hypothesis, ε0 ≤ 0.1, and alternative hypothesis, ε1 = 0.01, with a power of the not-close fit test = 0.8, model degrees of freedom = 10, and significance level of 5%. Accordingly, we determined that the minimum required sample size was 177 [34]. Statistical significance was considered at the 5% level when the between-parameter difference in the test statistic was ≥1.96. The scores for dietary salt restriction practices, the dietary Na/K ratio, salt intake, BMI, and frequency of eating out were considered as interval scales, with sex and age group differences being examined using a two-way analysis of variance. Furthermore, we confirmed the absence of interaction effects among them. Multiple comparisons using Tukey’s honestly significant difference test were performed if a main effect was found for the age group. Statistical significance was set at *p* < 0.05. Statistical analyses were performed using IBM SPSS Statistics for Windows, version 29.0 (IBM Corp., Armonk, NY, USA).

## 3. Results

Table 1 shows the participant characteristics according to sex. Obesity prevalence (BMI ≥ 25 kg/m^2^) was lower among women (18.4%) than among men (29.5%; *p* = 0.004). In terms of vegetable and fruit intake, women were more likely than men to consume three or more vegetable dishes daily (50.0% vs. 33.7%) (*p* = 0.002), and women reported higher vegetable (176.0 vs. 139.5 g per 1000 kcal) and fruit (64.3 vs. 35.1 g per 1000 kcal) intakes (both *p* < 0.001).

Table 2 compares dietary-related factors according to the presence/absence of dietary salt restriction practices among middle-aged and older Japanese adults for men and women. Dietary salt restriction was associated with a higher potassium intake (men: *p* = 0.009, women; *p* < 0.001) and lower Na/K ratios for both sexes (men: *p* = 0.008, women; *p* = 0.002). Moreover, it was associated with a higher protein/energy ratio (*p* = 0.033) and higher fruit intake (*p* = 0.013) for men and higher vegetable intake (*p* = 0.007) for women.

Table 3 compares BMI-related eating and lifestyle behaviours according to the presence/absence of obesity among middle-aged and older Japanese adults for each sex. Dietary and lifestyle behaviours did not differ significantly according to the presence/absence of obesity. However, for the items included in the initial hypothetical model, obese men tended toward higher eating out frequency (*p* = 0.064) and obese women tended toward higher use of home meal replacement (*p* = 0.061).

**Figure 2:**
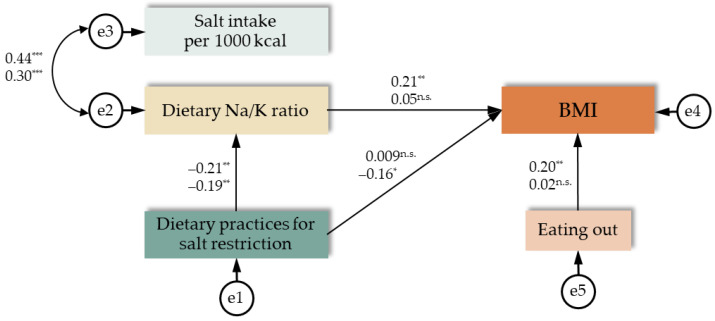
Associations of dietary salt restriction practices with the dietary sodium/potassium (Na/K) ratio, salt intake, and body mass index (BMI) (*n* = 418). Numbers in the path diagram next to the straight arrows indicate standardised estimates, and numbers in the path diagram next to the two-way arc arrows indicate correlation coefficients. Statistical significance was set at * *p* < 0.05, ** *p* < 0.01, and *** *p* < 0.001 (n.s., not significant). Each pair’s upper and lower numbers are for men (*n* = 190) and women (*n* = 228), respectively. The results of simultaneous multi-population analysis according to sex suggest that the hypothesised model, excluding home meal replacement use, has a more optimal fit [χ^2^ = 8.771; *df* = 10 (*p* = 0.554); GFI = 0.992; AGFI = 0.975; CFI = 1.000; RMSEA < 0.001; AIC = 48.771].

We analysed an initial hypothetical model (Figure 1) of factors involved in dietary salt restriction practices. The model’s goodness-of-fit indices were somewhat less than acceptable (GFI = 0.975; AGFI = 0.941; CFI = 0.870; RMSEA = 0.045; AIC = 80.989). Therefore, we established a better-fit model by deleting home meal replacement use from the initial model. Figure 2 shows the results of a simultaneous multi-population analysis for each sex, evaluating the associations of dietary salt restriction practices with the dietary Na/K ratio, salt intake, and BMI, leaving only the frequency of eating out in the hypothetical model.

Dietary salt restriction was associated with a low dietary Na/K ratio (standardised estimate: –0.21, *p* = 0.001 for men; –0.19, *p* = 0.002 for women) for both sexes. Dietary Na/K ratio showed a significant and positive correlation with salt intake per 1000 kcal (correlation coefficient: 0.44, *p* < 0.001 for men; 0.30, *p* < 0.001 for women) in both sexes. Dietary salt restriction was associated with lower values of BMI among men (0.21, *p* = 0.004), mediated by the Na/K ratio; the association was relatively weak among women (–0.16, *p* = 0.018). For men, eating out frequency was associated with higher values of BMI (0.20, *p* = 0.005).

Table 4 shows the differences in dietary salt restriction practices and the related factors according to sex and age groups among middle-aged and older Japanese adults.

Dietary salt restriction practices scores were significantly higher in women than in men [*F*(1412) = 21.713, *p* < 0.001], and in individuals aged 60–69 years than in those aged 40–49 years [*F*(2412) = 7.530, *p* = 0.001]. The dietary Na/K ratio was significantly lower in women than in men [*F*(1412) = 389.280, *p* < 0.001] and in individuals aged 50–59 and 60–69 years than in those aged 40–49 years [*F*(2412) = 5.364, *p* = 0.005]. Salt intake (per 1000 kcal) was significantly higher in women than in men [*F*(1412) = 69.303, *p* < 0.001], whereas BMI [*F*(1412) = 23.567, *p* < 0.001] and the eating out frequency score [*F*(1412) = 6.143, *p* = 0.014] were significantly higher in men. No significant interactions between sex and age group were observed in these scores/values.

## 4. Discussion

This study investigated the structural associations of dietary behaviours, such as restricting salt intake and consumption of potassium-rich fruits and vegetables, with obesity and hypertension prevention in middle-aged and older Japanese adults at increased risk of obesity and hypertension.

In the Japanese daily dietary survey, vegetables and fruits were the greatest sources of potassium intake [35]. In the present study, we also found that a low salt intake resulting from a higher vegetable and fruit intake was associated with a lower BMI. Foods with low caloric density occupy a greater stomach volume than foods with high caloric density because they contain more fibre and water, resulting in low overall caloric intake and quick satiety [36]. Polyphenols [37], carotenoids [38], and vitamin C [39], which are found in fruits and vegetables, may help reduce body weight and BMI. Nevertheless, a systematic review and meta-analysis [20] proposed reasons for the insufficient evidence for high vegetable and fruit intake resulting in weight loss. Additionally, the US Department of Agriculture reported an increase in fruit and vegetable consumption from 1970 to 2018; however, a concomitant increase occurred in the consumption of meat, eggs, nuts, and grains, which may be problematic [40]. Therefore, as previously noted [20], it is difficult to lose weight and prevent obesity without combining recommendations for vegetable and fruit intake with guidance on healthy cooking methods and foods choices with a lower caloric density (high fibre and water content). In the current study, we observed no difference in the daily energy intake according to the presence/absence of dietary salt restriction practices. In Japan, ‘Specific Health Checkups and Specific Health Guidance’ [41] have been provided since FY2008 for people aged 40–74 years to prevent or improve metabolic syndrome (visceral fat syndrome). Additionally, health guidance is provided according to the observed degree of visceral fat accumulation and the number of risk factors, including high blood pressure, dyslipidaemia, and high blood sugar levels. Individuals attempting to reduce their salt intake should be informed about controlling their energy intake and eating sufficient vegetables and fruits. This point is consistent with our finding that adopting dietary salt restriction practices was associated with a low BMI via the dietary Na/K ratio among men, considering that obesity was more prevalent among men.

The direct correlation between dietary salt restriction practices and lower values of BMI among women, with no mediation by the dietary Na/K ratio, may be attributed to the lower proportion of obese individuals in the subgroup of women and the higher proportion of women practising dietary salt restriction (68%), given the high prevalence [42] of hypertension among post-menopausal women. In addition, vegetable intake was higher among women who practised salt restriction. The dietary Na/K ratio was extremely low for women compared with that for men. Furthermore, even women without dietary salt restriction practices had a lower dietary Na/K ratio than men who practised salt restriction. Accordingly, women demonstrated a direct association between dietary salt restriction practices and a low BMI without mediation by the dietary Na/K ratio, which can be achieved by combining sodium reduction and consumption of potassium-rich fruits and vegetables. Nonetheless, higher vegetable and fruit intakes and a low dietary Na/K ratio were consistently associated with a low BMI among individuals practising dietary salt restriction, regardless of sex. The negative effect of eating out on the relationship between dietary salt restriction practices and low BMI via the dietary Na/K ratio in men may be attributed to the positive correlation between salt intake and the frequency of eating out [43] and the association between eating out and BMI [26,27]. In the present study, men with obesity only tended to eat out more frequently, which was not a significant difference. Nevertheless, structural analysis of covariance revealed that the frequency of eating out was associated with BMI via the dietary Na/K ratio from dietary salt restriction practices. The lack of a negative effect of eating out among women could be attributed to the low prevalence of obesity and high rate of dietary salt restriction practices compared with that among men, with many women being aware that eating out can lead to obesity [26,27] and high salt intake [43].

Despite its contributions, this study has some limitations. First, the participants in this study were residents of one region in Japan, Hyogo Prefecture, and were a highly health-conscious population who cooperated in the 2021 Hyogo Nutrition and Diet Survey. Additionally, because this study was a cross-sectional, we could not elucidate the causal relationship between dietary salt restriction and decreased BMI. A longitudinal study should be conducted to clarify the effect of dietary salt reduction practices and the accompanying dietary Na/K ratio on BMI and body weight. Second, this study’s categorisation of dietary salt restriction practices was based on participant awareness, not the amount of dietary salt restriction. Third, the energy/nutrient and food group intakes calculated using data from the short-FFQ are less accurate than those computed using data from multi-day food records or 24 h dietary recalls. Nonetheless, the short-FFQ can be considered a valid assessment tool for determining the dietary Na/K ratio given its moderate correlation with the estimated quantities obtained from 12-day weighed food records [32]. In the future, we intend to examine the effects of dietary salt restriction practices and Na/K ratio on BMI through follow-up studies addressing a larger sample of Japanese individuals. Fourth, the items related to salt reduction practices and eating habits used in this study were not subjected to validity studies. However, the same items were used in a survey conducted by the Ministry of Education, Culture, Sports, Science and Technology in Japan.

## 5. Conclusions

Our study revealed that dietary salt restriction practices in middle-aged and older Japanese adults may contribute to improved hypertension and overweight/obesity prevention, and dietary Na/K ratio may play a role as a mediating factor in men. For men, eating out was also associated with higher values of BMI. With respect to future policies, the food service industry should be actively encouraged to promote the strengthening of healthy eating environments by offering low-salt meals with ample vegetables and fruits. Furthermore, the causal relationship of salt restriction and vegetable and fruit intake with obesity prevention needs to be further investigated by longitudinal studies and studies involving non-Japanese populations and be recognised by the general public.

## Figures and Tables

**Table 1:** Participant characteristics (*n* = 418).

Characteristics	Total		Men		Women		
	*n* = 418		*n* = 190		*n* = 228		*p*
	*n*	%	*n*	%	*n*	%	
Age ^a^							
40 to 49 years	131	31.3	65	34.2	66	28.9	0.20
50 to 59 years	114	27.3	44	23.2	70	30.7	
60 to 69 years	173	41.4	81	42.6	92	40.4	
Living Arrangement ^a^							
Living alone	34	8.1	15	7.9	19	8.3	0.89
Married couple	110	26.3	48	25.3	62	27.2	
Parent(s) and children	216	51.7	102	53.7	114	50.0	
3- or 4-generation household	54	12.9	24	12.6	30	13.2	
Others	4	1.0	1	0.5	3	1.3	
Body mass index (BMI, kg/m^2^) ^a^							
<18.5	17	4.1	3	1.6	14	6.1	0.004
≥18.5 and <25	303	72.5	131	68.9	172	75.4	
≥25	98	23.4	56	29.5	42	18.4	
Number of vegetable dishes daily ^a^							
5 dishes or more	21	5.0	4	2.1	17	7.5	0.002
4 dishes	39	9.3	12	6.3	27	11.8	
3 dishes	118	28.2	48	25.3	70	30.7	
2 dishes	163	39.0	81	42.6	82	36.0	
1 dish or less	77	18.4	45	23.7	32	14.0	
Vegetable intake (g/1000 kcal) ^b^							
	168.2		139.5		176.0		<0.001
	141.3	179.6	127.7	157.1	168.8	184.0	
Fruit intake (g/1000 kcal) ^b^	58.8						
	36.1	66.0	35.1		64.3		<0.001
			30.6	40.9	59.9	70.7	

Values for vegetable and fruit intake are shown as median (upper line) and 25th and 75th percentile (lower line). ^a^ Chi-squared test. ^b^ Mann–Whitney U test.

**Table 2:** Comparison of dietary-related factors according to the presence/absence of dietary salt restriction practices among middle-aged and older Japanese adults (*n* = 418).

Dietary-Related Factors	Men, *n* = 190			Women, *n* = 228		
	Dietary Salt Restriction			Dietary Salt Restriction		
	Practising	Not Practising	*p*	Practising	Not Practising	*p*
	*n* = 81	*n* = 109		*n* = 155	*n* = 73	
	Mean	Mean		Mean	Mean	
	SD	SD		SD	SD	
Body mass index (BMI, kg/m^2^)	23.6	24.2	0.22	22.2	23.1	0.07
	2.7	3.4		3.2	3.7	
Energy intake (kcal)	2254	2272	0.65	1807	1806	0.93
	280	263		56	40	
Protein/energy ratio (%E)	15.0	14.7	0.033	15.5	15.4	0.08
	0.8	0.8		0.6	0.3	
Fat/energy ratio (%E)	23.4	23.1	0.25	24.5	24.4	0.08
	2.0	1.9		0.4	0.4	
Carbohydrate/energy ratio (%E)	50.7	51.0	0.51	54.2	54.3	0.37
	3.7	3.7		1.5	1.5	
Sodium intake (mg/1000 kcal)	1935	1936	0.97	2034	2032	0.87
	135	144		85	83	
Potassium intake (mg/1000 kcal)	1378	1336	0.009	1622	1581	*p* < 0.001
	119	91		104	62	
Dietary Na/K ratio (mmol/mmol)	2.40	2.47	0.008	2.14	2.19	0.002
	0.20	0.16		0.12	0.09	
Salt intake (g/1000 kcal)	4.9	4.9	0.98	5.1	5.1	0.83
	0.3	0.4		0.2	0.2	
Vegetable intake (g/1000 kcal)	150.1	143.1	0.11	183.2	176.5	0.007
	33.4	26.3		22.6	13.9	
Fruit intake (g/1000 kcal)	41.8	36.5	0.013	67.4	68.0	0.72
	16.6	11.0		12.7	14.4	

Values are shown as mean ± SD (standard deviation). Welch’s *t*-test was used.

**Table 3:** Comparison of body mass index (BMI)-related eating and lifestyle behaviours according to the presence/absence of obesity among middle-aged and older Japanese adults (*n* = 418).

BMI-Related Eating and Lifestyle Behaviours	Men, *n* = 190					Women, *n* = 228				
	Obese, BMI ≥ 25 kg/m^2^		Normal Weight/Underweight, BMI < 25 kg/m^2^		*p*	Obese, BMI ≥ 25 kg/m^2^		Normal Weight/Underweight, BMI < 25 kg/m^2^		*p*
	*n* = 56		*n* = 134			*n* = 42		*n* = 186		
	*n*	%	*n*	%		*n*	%	*n*	%	
Snacking frequency										
At least twice daily	4	7.1	10	7.5	0.74	3	7.1	35	18.8	0.48
6 or 7 days/week	10	17.9	29	21.6		20	47.6	61	32.8	
4 or 5 days/week	12	21.4	17	12.7		11	26.2	30	16.1	
2 or 3 days/week	17	30.4	38	28.4		6	14.3	36	19.4	
1 day/week or less	13	23.2	40	29.9		2	4.8	24	12.9	
Late-night snacking frequency										
At least twice daily	0	0.0	0	0.0	0.17	0	0.0	0	0.0	0.24
6 or 7 days/week	1	1.8	4	3.0		1	2.4	2	1.1	
4 or 5 days/week	5	8.9	1	0.7		1	2.4	2	1.1	
2 or 3 days/week	5	8.9	9	6.7		4	9.5	8	4.3	
1 day/week or less	45	80.4	120	89.6		36	85.7	174	93.5	
Drinking (alcohol) frequency										
Daily	17	30.4	49	36.6	0.88	2	4.8	18	9.7	0.73
5 or 6 days/week	6	10.7	12	9.0		2	4.8	5	2.7	
3 or 4 days/week	3	5.4	9	6.7		2	4.8	10	5.4	
1 or 2 days/week	6	10.7	8	6.0		2	4.8	9	4.8	
1–3 days/month	5	8.9	4	3.0		5	11.9	19	10.2	
Quit (have quit for more than 1 year)	2	3.6	3	2.2		3	7.1	1	0.5	
Hardly drink	17	30.4	49	36.6		26	61.9	124	66.7	
Eating out frequency										
At least twice daily	1	1.8	1	0.7	0.064	0	0.0	0	0.0	0.91
Once daily	0	0.0	2	1.5		0	0.0	0	0.0	
4 to 6 days/week	5	8.9	1	0.7		1	2.4	2	1.1	
2 or 3 days/week	7	12.5	9	6.7		3	7.1	12	6.5	
1 day/week	3	5.4	15	11.2		2	4.8	14	7.5	
1–3 days/month	31	55.4	74	55.2		25	59.5	117	62.9	
Not at all	9	16.1	32	23.9		11	26.2	41	22.0	
Home meal replacement (ready-to-eat food) frequency										
At least twice daily	0	0.0	1	0.7	0.48	0	0.0	0	0.0	0.061
Once daily	2	3.6	3	2.2		0	0.0	3	1.6	
4 to 6 days/week	2	3.6	7	5.2		2	4.8	7	3.8	
2 or 3 days/week	6	10.7	17	12.7		9	21.4	20	10.8	
1 day/week	13	23.2	15	11.2		8	19.0	20	10.8	
1–3 days/month	25	44.6	59	44.0		20	47.6	107	57.5	
Not at all	8	14.3	32	23.9		3	7.1	29	15.6	
Exercise regularity (including walking at least 30 min once)										
At least 3 times/week for at least 1 year	8	14.3	29	21.6	0.24	5	11.9	30	16.1	0.88
2 times/week for more than 1 year	7	12.5	21	15.7		6	14.3	33	17.7	
At least 2 times/week for less than 1 year	7	12.5	14	10.4		8	19.0	11	5.9	
Almost never	25	44.6	48	35.8		20	47.6	81	43.5	
Not at all	9	16.1	22	16.4		3	7.1	31	16.7	

Welch’s *t*-test was used, as the data on the factors were analysed as interval scales in the covariance structure analysis. Snacking and late-night snacking frequency interval scale: 5 = At least twice daily; 4 = 6 or 7 days/week; 3 = 4 or 5 days/week; 2 = 2 or 3 days/week; 1 = 1 day/week or less. Drinking frequency interval scale: 7 = Daily; 6 = 5 or 6 days/week; 5 = 3 or 4 days/week; 4 = 1 or 2 days/week; 3 = 1–3 days/month; 2 = Quit; 1 = Hardly drink. Eating out or home meal replacement frequency interval scale: 7 = At least twice daily; 6 = Once daily; 5 = 4 to 6 days/week; 4 = 2 or 3 days/week; 3 = 1 day/week; 2 = 1–3 days/month; 1 = Not using at all or not at all. Exercise regularity interval scale: 5 = At least 3 times/week for at least 1 year; 4 = 2 times/week for more than 1 year; 3 = At least 2 times/week for less than 1 year; 2 = Almost never; 1 = Not at all.

**Table 4:** Differences in dietary salt restriction practices and the related factors according to sex and age groups among middle-aged and older Japanese adults (*n* = 418).

	Men, *n* = 190			Women, *n* = 228			Interaction	Sex		Age Group	Multiple Comparison ^a^
	40–49 Years	50–59 Years	60–69 Years	40–49 Years	50–59 Years	60–69 Years					
	*n* = 65	*n* = 44	*n* = 81	*n* = 66	*n* = 70	*n* = 92	(Age × Sex)				
	Mean	Mean	Mean	Mean	Mean	Mean					
	SD	SD	SD	SD	SD	SD					
Dietary practices for salt restriction							*F* = 0.723	*F* = 21.713		*F* = 7.530	
Score (1–4 points)	2.18	2.43	2.57	2.67	2.67	3.01	*df* = 2412	*df* = 1412	Male < Female	*df* = 2412	40–49 < 60–69 **
	0.85	0.73	0.87	0.81	0.88	0.81	*p* = 0.49	*p* < 0.001		*p* = 0.001	
Dietary Na/K ratio (mmol/mmol)							*F* = 0.872	*F* = 389.280		*F* = 5.364	
	2.46	2.46	2.41	2.19	2.15	2.14	*df* = 2412	*df* = 1412	Male > Female	*df* = 2412	40–49 > 50–59
	0.19	0.16	0.18	0.09	0.11	0.12	*p* = 0.42	*p* < 0.001		*p* = 0.005	40–49 > 60–69 *
Salt intake (g/1000 kcal)							*F* = 0.634	*F* = 69.303		*F* = 0.591	
	4.89	4.88	4.89	5.10	5.12	5.16	*df* = 2412	*df* = 1412	Male < Female	*df* = 2412	
	0.37	0.38	0.34	0.21	0.20	0.22	*p* = 53	*p* < 0.001		*p* = 0.55	
Body mass index (BMI, kg/m^2^)							*F* = 1.559	*F* = 23.567		*F* = 0.702	
	23.8	24.4	23.9	22.2	22.1	23.0	*df* = 2412	*df* = 1412	Male > Female	*df* = 2412	
	3.3	2.8	3.1	3.7	3.2	3.2	*p* = 0.21	*p* < 0.001		*p* = 0.50	
Eating out frequency							*F* = 0.399	*F* = 6.143		*F* = 2.106	
Score (1–7 points)	2.31	2.41	2.09	2.09	2.04	1.93	*df* = 2412	*df* = 1412	Male > Female	*df* = 2412	
	1.00	1.24	1.20	0.76	0.92	0.80	*p* = 0.67	*p* = 0.014		*p* = 0.12	

Two-way ANOVA. ^a^ Tukey’s honestly significant difference test: *p* < 0.05, * *p* < 0.01, ** *p* < 0.001.

## Data Availability

The data are not publicly available due to confidentiality reasons.
